# Supplementary material for: Challenges and Opportunities of Universal Health Coverage in Africa: A Scoping Review
Source: Int J Environ Res Public Health. 2025 Jan 10;22(1):86. doi: 10.3390/ijerph22010086 (PMC11764768; doi:10.3390/ijerph22010086)
Supplement: Supplementary file 1 [file ijerph-22-00086-s001.zip › ijerph-3275163-Supplementary Table S1.pdf]

Supplementary Table S1: Characteristics of papers included in the scoping review on universal health coverage in Africa					
	**Article title**	**Author**	**Type of article**	**year**	**Geography**
(1)	What do we know about financial protection in health in Africa? A systematic review	Grace Achungura Kabaniha,Doris Osei Afriyie, Mayur L Mandalia, John E Ataguba	systematic review	2020	Africa-Multicountry
(2)	Challenges to Achieving Universal Health Coverage Throughout the World: A Systematic Review	Darrudi, Alireza, Mohammad Hossein Ketabchi Khoonsari, and Maryam Tajvar	systematic review	2022	Global-Africa included
(3)	Gabon gets everyone under one social health insurance roof	Gary Humphreys	Report	2013	Gabon
(4)	Towards universal health coverage in Zambia: impediments and opportunities	Carolien Aantjes,Timothy Quinlan &Joske Bunders	Qualitative study	2016	Zambia
(5)	Gaps in universal health coverage in Malawi: A qualitative study in rural communities	Gilbert Abotisem Abiiro, Grace Bongololo Mbera & Manuela De Allegri	Qualitative study	2014	Malawi
(6)	Universal health coverage necessitates a system approach: an analysis of Community-based Health Planning and Services (CHPS) initiative in Ghana	Assan A, Takian A, Aikins M, Akbarisari A.	Qualitative study	2018	Ghana
(7)	Nigeria National Health Insurance Scheme: A Highly Subsidized Health Care Program for a Privileged Few	Adewole, D.A. and Osungbade, K.O.,	Observational	2016	Nigeria

(8)	Pro–poor pathway towards universal health coverage: lessons from Ethiopia	Kesetebirhan Admasu1 , Taye Balcha1 , Tedros Adhanom Ghebreyesus2	case study	2016	Ethiopia
(9)	The “Universal” in UHC and Ghana’s National Health Insurance Scheme: policy and implementation challenges and dilemmas of a lower middle income country	Irene Akua Agyepong1* , Daniel Nana Yaw Abankwah2 , Angela Abroso3 , ChangBae Chun5 , Joseph Nii Otoe Dodoo4 , Shinye Lee5 , Sylvester A. Mensah6 , Mariam Musah6 , Adwoa Twum6 , Juwhan Oh7 , Jinha Park5 , DoogHoon Yang5 , Kijong Yoon8 , Nathaniel Otoo6 and Francis Asenso-Boadi6	Observational	2016	Ghana
(10)	Policy dialogue and participation: a new way of crafting a national health financing strategy in Morocco	El Houcine Akhnif1* , Hafid Hachri1 , Abdelouahab Belmadani2 , Awad Mataria3 and Maryam Bigdel	Observational	2020	Morocco
(11)	Advancing alternative health care financing through effective community partnership: A necessity for universal health coverage in Nigeria	Isaac Akintoyese Oyekola, John Olusegun Ojediran, Oludele Albert Ajani, Eyitayo Joseph Oyeyipo & Bamidele Rasak	Narrative Review	2020	Nigeria
(12)	Health Financing Systems, Health Equity and Universal Health Coverage in Arab Countries	Randa Alami	Narrative review	2017	Arab countries - Egypt and Tunisia in Africa

(13)	A Review of the National Health Insurance Scheme in Ghana: What Are the Sustainability Threats and Prospects?	Robert Kaba Alhassan1,2*, Edward Nketiah-Amponsah3 , Daniel Kojo Arhinful	systematic review	2016	Ghana
(14)	Financing universal health coverage: four steps to go from aspiration to action	Amir Aman, Diane Gashumba, Ira Magaziner,Anders Nordström	Editorial	2019	Global-Africa included
(15)	Health insurance and health system (un) responsiveness: a qualitative study with elderly in rural Tanzania	Paul Joseph Amani1,2*, Anna-Karin Hurtig2 , Gasto Frumence3 , Angwara Denis Kiwara3 , Isabel Goicolea2 and Miguel San Sebastiån	Qualitative study	2021	Tanzania
(16)	South Sudan’s road to universal health coverage: a slow but steady journey	Victoria Achut Anib1, Mayen Machut Achiek1, Fabian Ndenzako2, Olushayo Oluseun Olu	Editorial	2022	Sudan
(17)	Mundane? Demographic characteristics as predictors of enrolment onto the National Health Insurance Scheme in two districts of Ghana	Anthony Seddoh and Fuseini Sataru*	Observational	2018	Ghana
(18)	Universal health coverage still rare in Africa	Appiah, Bernard	commentary	2012	Rwanda and Ghana
(19)	A Review of Recent Developments and Future Challenges in the Implementation of Universal Health	Irfat Ara1 , Mehrukh Zehravi2 , Mudasir Maqbool3 * and Imran Gani3	commentary	2022	Global-Africa included

	Coverage Policy Framework in Some Countries				
(20)	Enhancing Political Will for Universal Health Coverage in Nigeria	Bolaji S. Aregbeshola	commentary	2017	Nigeria
(21)	Can a low income country move towards universal health coverage?	Sophie Arie	Report	2019	Mali
(22)	Challenges to achieving universal health coverage through communitybased health planning and services delivery approach: a qualitative study in Ghana	Abraham Assan,1,2 Amirhossein Takian,1,3,4 Moses Aikins,5 Ali Akbarisari1	Qualitative study	2019	Ghana
(23)	Assessing equitable health financing for universal health coverage: a case study of South Africa	John E. Ataguba	case study	2016	South Africa
(24)	Universal health coverage law approved in Egypt	Sharmila Devi	commentary	2018	Egypt
(25)	Monitoring Process Barriers and Enablers Towards Universal Health Coverage Within the Sustainable Development Goals: A Systematic Review and Content Analysis	Naser Derakhshani 1,2 Leila Doshmangir 1,3 Ayat Ahmadi 4 Ali Fakhri5 Homayoun SadeghiBazargani6 Vladimir Sergeevich Gordeev 7,	systematic review	2020	Global-Africa included

(26)	Effect of Community-Based Health Insurance on Utilization of Outpatient Health Care Services in Southern Ethiopia: A Comparative Cross-Sectional Study	Bekele Demissie 1 Keneni Gutema Negeri 2	Observational	2020	Ethiopia
(27)	Reforms for financial protection schemes towards universal health coverage, Senegal	Bocar Mamadou Daff,a Serigne Diouf,a Elhadji Sala Madior Diop,a Yukichi Mano,b Ryota Nakamura,c Mouhamed Mahi Sy,a Makoto Tobe,d Shotaro Togawad & Mor Ngom	Observational	2020	Senegal
(28)	Monitoring and Evaluating Progress towards Universal Health Coverage in Tunisia	Mohamed Kouni Chahed[1] and Chokri Arfa[2]	case study	2014	Tunisia
(29)	Can National Health Insurance Pave the Way to Universal Health Coverage in Sub-Saharan Africa?	Cheryl Cashin & Jean-Paul Dossou	Observational	2021	Sub-saharan Africa
(30)	The feasibility analysis of integrating community-based health insurance schemes into the national health insurance scheme in uganda	Prossy Kiddu Namyalo1* , Boniface Mutatina2 , Sarah Byakika3 , Aliyi Walimbwa 3 , Rose Kato4 , Robert K. Basaza5	Qualitative study	2023	Uganda
(31)	Health financing reforms for Universal Health Coverage in five emerging economies	Chris Atim1 , Indu Bhushan2 , Mark Blecher3 , Ramana Gandham4 , Vikram Rajan5 , Jonatan	case studies	2021	Kenya, Ghana, South Africa,

		Davén3 , Olusoji Adeyi			
(32)	Healthcare quality under the National Health Insurance Scheme in Ghana: perspectives from premium holders	Atinga, Roger Ayimbillah	Cross section	2012	Ghana
(33)	Path Dependence and Universal Health Coverage: The Case of Egypt	Ayman Fouda, Francesco Paolucci	case study	2017	Egypt
(34)	Kenya National Hospital Insurance Fund Reforms: Implications and Lessons for Universal Health Coverage	Barasa, E.; Rogo, K.; Mwaura, N.; Chuma, J.	Review	2018	Kenya
(35)	Universal Health Coverage and Social Health Protection: Policy relevance to health system financing reforms	Bayarsaikhan, D.; Tessier, L.; Ron, A.	policy analysis	2022	LMIC-Africa included
(36)	Universal access to healthcare: The case of South Africa in the comparative global context of the late anthropocene era	Benatar, S.; Gill, S.	Expert opinion	2020	South Africa
(37)	Health financing lessons from Thailand for South Africa on the path towards universal health coverage	Blecher, M.; Pillay, A.; Patcharanarumol, W.; Panichkriangkrai, W.; Tangcharoensathien, V.; Teerawattananon,	Case report	2016	South Africa

		Y.; Pannarunothai, S.; DavÃ©n, J.			
(38)	Promoting universal financial protection: a case study of new management of community health insurance in Tanzania	Borghi, J.; Maluka, S.; Kuwawenaruwa, A.; Makawia, S.; Tantau, J.; Mtei, G.; Ally, M.; Macha, J.	Case study	2013	Tanzania
(39)	Modelling the implications of moving towards universal coverage in Tanzania	Borghi, J.; Mtei, G.; Ally, M.	case study	2012	Tanzania
(40)	Making fair choices on the path to universal health coverage: Final report of the WHO Consultative Group on Equity and Universal Health Coverage	World Health Organization	Report	2014	Global
(41)	South Africa passes National Health Insurance Bill	Munyaradzi Makoni	commentary	2023	South Africa
(42)	Health worker resourcing to meet universal health coverage in Africa	Cerf, M. E.	Research article	2021	Africa
(43)	Innovative domestic financing mechanisms for health in Africa: An evidence review	Nouria Brikci	systematic review	2024	Africa

(44)	Universal Health Coverage: A burning need for developing countries	Zaman, Sojib Bin; Hossain, Naznin	Research article	2017	LMIC-Africa included
(45)	The political path to universal health coverage: Power, ideas and community-based health insurance in Rwanda	Chemouni, Benjamin	Case study	2018	Rwanda
(46)	Crunch Time: The Transformational Universal Health Coverage Agenda for Zambia	Chilufya, C.; Kamanga, M.	case study	2018	Zambia
(47)	Implementation of the national health insurance scheme (NHIS) in Ghana: Lessons for south africa and low-and middle-income countries	Christmals, C. D.; Aidam, K.	Scoping review	2020	Ghana
(48)	Does the distribution of health care benefits in Kenya meet the principles of universal coverage?	Chuma, J.; Maina, T.; Ataguba, J.	Observational	2012	Kenya
(49)	Contributions and challenges of healthcare financing towards universal health coverage in Ethiopia: a narrative evidence synthesis	Debie, A.; Khatri, R. B.; Assefa, Y.	Narrative Review	2022	Ethiopia

(50)	An appraisal of the national health insurance scheme of Nigeria	Monye FN.	Cross section	2006	Nigeria
(51)	Ghana's National Health Insurance Scheme: helping the poor or leaving them behind?	Dixon, Jenna; Tenkorang, Eric Y; Luginaah, Isaac	Cross section	2011	Ghana
(52)	Does expanding fiscal space lead to improved funding of the health sector in developing countries?: lessons from Kenya, Lagos State (Nigeria) and South Africa	Doherty, J.; Kirigia, D.; Okoli, C.; Chuma, J.; Ezumah, N.; Ichoku, H.; Hanson, K.; McIntyre, D.	Mixed methods	2018	Kenya, South Africa, and Nigeria
(53)	Adopting localised health financing models for universal health coverage in Low and middle-income countries: lessons from the National Health Insurance Scheme in Ghana	Domapielle, M. K.	Narrative Review	2021	Ghana
(54)	Universal Health Coverage in low- and middle-income countries: Lessons learned and way forward	Dzekem, Bonaventure	policy brief	2018	Cameroon
(55)	Strategic Purchasing Arrangements in Uganda and Their Implications for Universal Health Coverage	Ekirapa-Kiracho, E.; Ssennyonjo, A.; Cashin, C.; Gatome-Munyua, A.; Olalere, N.; Ssempala, R.; Mayora, C.; Ssengooba, F.	review	2022	Uganda

(56)	National health systems strengthening as the primary strategy to achieve universal health coverage in african countries	Elhadi, Y. A. M.; Adebisi, Y. A.; Abel, U. V.; Daniel, E. M.; Zaghloul, A.; Lucero-Prisno, D. E., III	Narrative Review	2021	Rwanda, Kenya, Nigeria, Tanzania, Ghana, Tunisia, Democratic Republic of Congo, Zambia, Egypt and South Africa
(57)	Measuring progress towards universal health coverage: National and subnational analysis in Ethiopia	Eregata, G. T.; Hailu, A.; Memirie, S. T.; Norheim, O. F.	cross-sectional study	2019	Ethiopia
(58)	Strategic Health Purchasing in Nigeria: Investigating Governance and Institutional Capacities within Federal Tax-Funded Health Schemes and the Formal Sector Social Health Insurance Programme	Ezenwaka, U.; Gatome-Munyua, A.; Nwankwor, C.; Olalere, N.; Orji, N.; Ewelike, U.; Uzochukwu, B.; Onwujekwe, O.	Qualitative study	2022	Nigeria
(59)	Strategies for financing social health insurance schemes for providing universal health care: a comparative analysis of five countries	Fenny, A. P.; Yates, R.; Thompson, R.	systematic review	2021	Ghana, Ethiopia, Kenya, Rwanda and Tanzania
(60)	The national health insurance scheme in Ghana: Implementation challenges and proposed solutions	Gajate-Garrido, Gissele; Owusua, Rebecca	cross-sectional study	2013	Ghana

(61)	Point of view: The importance of leadership towards universal health coverage in low income countries	Gonani, A.; Muula, A. S.	commentary	2015	Malawi
(62)	Democratization and Universal Health Coverage: A Case Comparison of Ghana, Kenya, and Senegal	GrÃ©pin, Karen A; Dionne, Kim Yi	Case study	2013	Kenya, Ghana, Senegal
(63)	Strategic purchasing: The neglected health financing function for pursuing universal health coverage in low- and middle-income countries: Comment on â€œwhatâ€™s needed to develop strategic purchasing in healthcare? Policy lessons from a realist reviewâ€	Hanson, K.; Barasa, E.; Honda, A.; Panichkriangkrai, W.; Patcharanarumol, W.	commentary	2019	Africa
(64)	The Nigeria national health insurance authority act and its implications towards achieving universal health coverage	Ipinnimo, T. M.; Durowade, K. A.; Afolayan, C. A.; Ajayi, P. O.; Akande, T. M.	Narrative Review	2022	Nigeria
(65)	The rocky road to universal health coverage in Egypt: A political economy of health insurance reform from 2005â€“15	Ismail, S. A.	Narrative Review	2018	Egypt

(66)	Does the operations of the National Health Insurance Scheme (NHIS) in Ghana align with the goals of Primary Health Care? Perspectives of key stakeholders in northern Ghana	John Koku Awoonor-Williams, Paulina Tindana Philip Ayizem Dalinjong Harry Nartey James Akazili	cross-sectional study	2016	Ghana
(67)	Assessing the efficiency of countries in making progress towards universal health coverage: A data envelopment analysis of 172 countries	Jordi, E.; Pley, C.; Jowett, M.; Abou Jaoude, G. J.; Haghparast-Bidgoli, H.	Quantitative study	2020	Global-Africa included
(68)	Political settlements and pathways to universal health coverage	Kelsall, Tim; Hart, Tom; Laws, Edward	Mixed methods	2016	Democratic Republic of Congo
(69)	A Critical Analysis of Purchasing Arrangements in Kenya: The Case of the National Hospital Insurance Fund	Kenneth Munge, Stephen Mulupi Edwine W. Barasa Jane Chuma	case study	2017	Kenya
(70)	Policy learning and Universal Health Coverage in low- And middle-income countries	KiendrÃbÃogo, J. A.; De Allegri, M.; Meessen, B.	Qualitative study	2020	LMIC-Africa included
(71)	The Landscape of Strategic Health Purchasing for Universal Health Coverage in Burkina Faso: Insights from Five Major Health	KiendrÃbÃogo, J. A.; Tapsoba, C.; Kafando, Y.; KaborÃ©, I.; Sory, O.; YamÃ©ogo, S. P.	Qualitative study	2022	Burkina Faso

	Financing Schemes				
(72)	Reinforcing locally led solutions for universal health coverage: A logic model with applications in Benin, Namibia and Uganda	KiendrÃbÃogo, J. A.; Thoumi, A.; Mangam, K.; TourÃ©, C.; Mbaye, S.; Odero, P.; Owino, E.; Jones, C.; Kiwanuka, G. S.; Audi, Z.; Bloom, D.; Kinter, A.; Gamble Kelley, A.	Qualitative study	2021	Benin, Namibia and Uganda
(73)	The failure of community-based health insurance schemes in Tanzania: opening the black box of the implementation process	Kigume, R.; Maluka, S.	Qualitative study	2021	Tanzania
(74)	Electronic medical record systems: A pathway to sustainable public health insurance schemes in sub-Saharan Africa	Kiri, V. A.; Ojule, A. C.	literature review	2020	Sub-saharan Africa
(75)	Community and informal care providers at the heart of universal health coverage in sub-saharan africa: A position paper	Kisangala, E.; Mbivnjo, E. L.; Okeah, B. O.; Khan, M. A.	Position paper	2021	Africa

(76)	Best practices in achieving universal health coverage: a scoping review	Koohpayehzadeh, Jalil; Azami-Aghdash, Saber; Derakhshani, Naser; Rezapour, Aziz; Kalajahi, Riaz Alaei; Khasraghi, Javad Sajjadi; Nikoomanesh, Mahdi; Sabetrohani, Hamideh; Soleimanpour, Samira	Scoping review	2020	Global-Africa included
(77)	National health insurance fund (NHIF) in tanzania as a tool for improving universal coverage and accessibility to health care services	KUMBURU, Paschal Nathan	Mixed methods	2015	Tanzania
(78)	Alternative financing strategies for universal health coverage	Kutzin, Joseph; Yip, Winnie; Cashin, Cheryl	Narrative Review	2016	LMIC-Africa included
(79)	The impact of health insurance enrollment on health outcomes in Kenya	Mercy G Mugo	Quantitative study	2023	Kenya
(80)	What alternative and innovative domestic methods of healthcare financing can be explored to fix the current claims reimbursement challenges by the National Health Insurance Scheme of Ghana? Perspectives of health managers	Laar, A. S.; Asare, M.; Dalinjong, P. A.	Qualitative study	2021	Ghana

(81)	Investigating sustainability challenges for the National Health Insurance Fund in Tanzania: a modelling approach	Brianna Osetinsky ,1,2 Günther Fink,1,2 August Kuwawenaruwa ,3 Fabrizio Tediosi 1,	Quantitative study	2023	Tanzania
(82)	Effects of Health Insurance on Quality of Care in Low-Income Countries: A Systematic Review	Doris Osei Afriyie1,2 *, Brendan Kwesiga3† , Grace Achungura4 , Fabrizio Tediosi 1,2 and Günther Fink 1,2	systematic review	2023	LMIC-Africa included
(83)	Moving towards universal health coverage: Health insurance reforms in nine developing countries in Africa and Asia	Lagomarsino, G.; Garabrant, A.; Adyas, A.; Muga, R.; Otoo, N.	Narrative Review	2012	Africa
(84)	Aiming for Universal Health Coverage through insurance in Ethiopia: State infrastructural power and the challenge of enrolment	Lavers, T.	case study	2021	Ethiopia
(85)	Leave No One Behind in Middle-Income Countries. A Review of Progress and Policies	Lay, Jann; Priebe, Jan	Narrative Review	2021	Africa included
(86)	[On the development of health insurance in low-income countries: the case of African countries]	Letourmy, Alain	case study	2008	Africa

(87)	Towards universal health coverage: an evaluation of Rwanda Mutuelles in its first eight years	Lu, C.; Chin, B.; Lewandowski, J. L.; Basinga, P.; Hirschhorn, L. R.; Hill, K.; Murray, M.; Binagwaho, A.	Quantitative study	2012	Rwanda
(88)	Universal health coverage for inclusive and sustainable development: a synthesis of 11 country case studies	Maeda, Akiko; Araujo, Edson; Cashin, Cheryl; Harris, Joseph; Ikegami, Naoki; Reich, Michael R	case study	2014	Ethiopia and Ghana
(89)	Transforming Health Financing Systems in the Arab World Toward Universal Health Coverage	Mataria, Awad; El-Saharty, Sameh; Hamza, Mariam M; Hassan, Hoda K	scoping review	2021	Arab countries-Sudan, Morocco, Tunisia, Egypt
(90)	Accreditation as a path to achieving universal quality health coverage	Mate, K. S.; Rooney, A. L.; Supachutikul, A.; Gyani, G.	narrative review	2014	LMIC-Africa included
(91)	Improving health system quality in low-and middle-income countries that are expanding health coverage: A framework for insurance	Mate, K. S.; Sifrim, Z. K.; Chalkidou, K.; Cluzeau, F.; Cutler, D.; Kimball, M.; Morente, T.; Smits, H.; Barker, P.	Narrative Review	2013	LMIC-Africa included
(92)	Do private health providers help achieve Universal Health Coverage? A scoping review of the evidence from low-income countries	Laura Coveney1 , David Musoke 2 and Giuliano Russo	Scoping review	2023	LMIC-Africa included
(93)	Is universal coverage via social health insurance financially feasible in Swaziland?	Mathauer, I.; Musango, L.; Sibandze, S.; Mthethwa, K.; Carrin, G.	Quantitative study	2011	Swaziland

(94)	Moving towards universal health coverage: Strengthening the evidence ecosystem for the South African health system	Mathews, C.; Goga, A.; Loveday, M.; Zembe, W.; Daviaud, E.; Siegfried, N.; Lewin, S.	case study	2019	South Africa
(95)	Examining purchasing reforms towards universal health coverage by the National Hospital Insurance Fund in Kenya	Mbau, R.; Kabia, E.; Honda, A.; Hanson, K.; Barasa, E.	case study	2020	Kenya
(96)	Legal and institutional foundations for universal health coverage, Kenya	Mbindyo, R.; Kioko, J.; Siyoi, F.; Cheruiyot, S.; Wangai, M.; Onsongo, J.; Omwoyo, A.; Kisia, C.; Miriti, K.	policy analysis	2020	Kenya
(97)	What healthcare financing changes are needed to reach universal coverage in South Africa?	McIntyre, D.	Editorial	2012	South Africa
(98)	Challenges in financing universal health coverage in sub-Saharan Africa	McIntyre, Diane; Obse, Amarech G; Barasa, Edwine W; Ataguba, John E	Expert opinion	2018	Sub-saharan Africa
(99)	Social or national health insurance: pooling of resources and purchasing of health care	McIntyre, Di; Van den Heever, Alex	book chapter	2007	South Africa

(100)	The state and significant drivers of health systems efficiency in Africa: A systematic review and meta-analysis	Juliet Nabyonga-Orem1,2* , Christmals Christmal2 * , Kingsley F Addai3 , Kasonde Mwinga4 , Kizito Aidam5 , Gilbert Nachinab6 , Sylivia Namuli4 , James A Asamani	systematic review	2023	Africa
(101)	Universal Health Coverage, Gender Equality and Social Protection: A Health Systems Approach	Gita sen, veloshnee govender and salma el-gamal	Scoping review	2018	Africa included
(102)	Promoting universal financial protection: Evidence from seven low- and middle-income countries on factors facilitating or hindering progress	McIntyre, D.; Ranson, M. K.; Aulakh, B. K.; Honda, A.	case studies	2013	Tanzania, Costa Rica, Nigeria, Malawi, India, Georgia,Thailan d
(103)	Uptake of health insurance in Malawi in 2019-2020: Evidence from the Multiple Indicator Cluster Survey	Wingston Felix Ng'ambi1,2,3*, Farai Chigaru1 , Takondwa Mwase1 , Agnes Jack Banda4 , Joseph Mfutso-Beng	Cross section	2022	Malawi
(104)	Health for all: The journey to universal health coverage	Medcalf, Alexander James; Bhattacharya, Sanjoy; Momen, Hooman; Saavedra, Monica Alexandra; Jones, Margaret	report	2015	South Africa
(105)	The political economy of health financing reforms in Zimbabwe: a scoping review	Mhazo, A. T.; Maponga, C. C.	Scoping review	2022	Zimbabwe

(106)	Progress towards universal coverage: The health systems of Ghana, South Africa and Tanzania	Mills, A.; Ally, M.; Goudge, J.; Gyapong, J.; Mtei, G.	case study	2012	Ghana, South Africa, Tanzania
(107)	Equity in financing and use of health care in Ghana, South Africa, and Tanzania: implications for paths to universal coverage	Mills, A.; Ataguba, J. E.; Akazili, J.; Borghi, J.; Garshong, B.; Makawia, S.; Mtei, G.; Harris, B.; Macha, J.; Meheus, F.; McIntyre, D.	Cross section	2012	Ghana, South Africa, Tanzania
(108)	Right to health: (in) congruence between the legal framework and the health system	Mitano, F.; Ventura, C. A.; de Lima, M. C.; Balegamire, J. B.; Palha, P. F.	document analysis	2016	Mozambique
(109)	Making fair choices on the path to universal health coverage: a précis	Voorhoeve, Alex, Trygve Ottersen, and Ole F. Norheim	Research article	2016	Global
(110)	Monitoring and evaluating progress towards Universal Health Coverage in Tanzania	Mtei, G.; Makawia, S.; Masanja, H.	case study	2014	Tanzania
(111)	A new universalism? Universal health coverage and debates about rights, solidarity and inequality in Kenya	Muinde, J. V. S.; Prince, R. J.	Qualitative study	2022	Kenya
(112)	Health Coverage and Financial Protection in Uganda: A Political Economy Perspective	Nannini, M.; Biggeri, M.; Putoto, G.	Qualitative study	2022	Uganda

(113)	Unlocking the power of communities to achieve universal health coverage in Africa	Ndejjo, R.; Musinguzi, G.; Musoke, D.; Wanyenze, R. K.	Position paper	2021	Africa
(114)	Political economy and the pursuit of universal health coverage in Ghana: a case study of the National Health Insurance Scheme	Novignon, J.; Lanko, C.; Arthur, E.	Qualitative study	2021	Ghana
(115)	Role of Social Health Insurance towards Universal Health Coverage for East African Community: An Analytical Review	Nshimirimana, Desire Aime; Kokonya, Donald; Gitaka, Jesse; Nzioki, Japheth Mativo; Wesonga, Bernard O; Mwaura, Peter N; Ponoth, Premanand	Narrative Review	2021	East Africa Community
(116)	Towards the achievement of universal health coverage in the Democratic Republic of Congo: does the Country walk its talk?	Nyamugira, A. B.; Richter, A.; Furaha, G.; Flessa, S.	Quantitative study	2022	Democratic Republic of Congo
(117)	Universal health coverage in Rwanda: dream or reality	Nyandekwe, M.; Nzayirambaho, M.; Baptiste Kakoma, J.	Quantitative study	2014	Rwanda
(118)	Monitoring and evaluating progress towards Universal Health Coverage in Ghana	Nyonator, F.; Ofosu, A.; Segbafah, M.; d'Almeida, S.	summary	2014	Ghana
(119)	Exploring effectiveness of different health financing mechanisms in Nigeria; what needs to change	Obinna Onwujekwe, Nkoli Ezumah Chinyere Mbachu Felix Obi Hyacinth Ichoku Benjamin Uzochukwu Hong Wang	Qualitative study	2019	Nigeria

	and how can it happen?				
(120)	Progress in the face of cuts: A qualitative Nigerian case study of maintaining progress towards universal health coverage after losing donor assistance	Obi, U. S.; Ogbuoji, O.; Mao, W.; Shahid, M.; Onwujekwe, O.; Yamey, G.	Qualitative study	2021	Nigeria
(121)	Universal Health Coverage in Uganda: The critical health infrastructure, healthcare coverage and equity	Odokonyero, Tonny; Mwesigye, Francis; Adong, Annet; Mbowa, Swaibu	case study	2017	Uganda
(122)	Operationalizing universal health coverage in Nigeria through social health insurance	Okpani, A. I.; Abimbola, S.	Position paper	2015	Nigeria
(123)	Towards universal coverage: a policy analysis of the development of the National Health Insurance Scheme in Nigeria	Onoka, C. A.; Hanson, K.; Hanefeld, J.	policy analysis	2015	Nigeria
(124)	Promoting universal financial protection: constraints and enabling factors in scaling-up coverage with social health	Onoka, C. A.; Onwujekwe, O. E.; Uzochukwu, B. S.; Ezumah, N. N.	Qualitative study	2013	Nigeria

	insurance in Nigeria				
(125)	Health financing reform in Uganda: How equitable is the proposed National Health Insurance scheme?	Orem, Juliet Nabyonga; Zikusooka, Charlotte Muheki	Qualitative study	2010	Uganda
(126)	Governance factors that affect the implementation of health financing reforms in Tanzania: An exploratory study of stakeholders' perspectives	Osei Afriyie, D.; Hooley, B.; Mhalu, G.; Tediosi, F.; Mtenga, S. M.	Qualitative study	2021	Tanzania
(127)	Making universal health coverage effective in low- and middle-income countries: a blueprint for health sector reforms	Otieno, Peter O; Asiki, Gershim	Position paper	2020	LMIC-Africa included
(128)	Towards a sustainable health care financing in Ghana: Is the National Health Insurance the solution?	Owusu-Sekyere, Ebenezer; Bagah, Daniel A	Qualitative study	2014	Ghana
(129)	How is equity approached in universal health coverage? An analysis of global and country policy documents in Benin and Senegal	Paul, E.; Deville, C.; Bodson, O.; SambiÃ©ni, N. E.; Thiam, I.; Bourgeois, M.; Ridde, V.; Fecher, F.	document analysis	2019	Benin and Senegal

(130)	Universal health coverage in francophone Sub-Saharan Africa: Assessment of global health experts' confidence in policy options	Paul, E.; Fecher, F.; Meloni, R.; Van Lerberghe, W.	Qualitative study	2018	West Africa
(131)	An assessment of the core capacities of the Senegalese health system to deliver Universal Health Coverage	Paul, E.; Ndiaye, Y.; Sall, F. L.; Fecher, F.; Porignon, D.	Qualitative study	2020	Senegal
(132)	Structural Factors Responsible for Universal Health Coverage in Low- and Middle-Income Countries: Results From 118 Countries	Ranabhat, C. L.; Jakovljevic, M.; Dhimal, M.; Kim, C. B.	Cross section	2020	LMIC-Africa included
(133)	Contracting non-state providers for universal health coverage: Learnings from Africa, Asia, and Eastern Europe	Rao, K. D.; Paina, L.; Ingabire, M. G.; Shroff, Z. C.	Qualitative study	2018	Tanzania, Ghana, Bosnia, Bangladesh, South Africa, and Afghanistan
(134)	Progress towards universal health coverage in BRICS: Translating economic growth into better health	Rao, K. D.; Petrosyan, V.; Araujo, E. C.; McIntyre, D.	Narrative Review	2014	Brazil, the Russian Federation, India, China and South Africa
(135)	Achieving Universal Health Coverage (UHC): Dominance analysis across 183 countries highlights importance of strengthening health workforce	Reid, M.; Gupta, R.; Roberts, G.; Goosby, E.; Wesson, P.	Quantitative study	2020	LMIC-Africa included

(136)	Endorsement of universal health coverage financial principles in Burkina Faso	Isabelle Agier a , Antarou Ly b , Kadidiatou Kadio b , Seni Kouanda b , Valery Ridde	Mixed methods	2016	Burkina Faso
(137)	Street-level workers' criteria for identifying indigents to be exempted from user fees in Burkina Faso	Ridde, V.; Sombie, I.	Qualitative study	2012	Burkina Faso
(138)	Contributing to collaborative health governance in Africa: a realist evaluation of the Universal Health Coverage Partnership	Robert, E.; Zongo, S.; Rajan, D.; Ridde, V.	realist evaluation	2022	Burkina Faso Cabo Verde Liberia Niger Togo DRC
(139)	Universal health coverage, economic slowdown and system resilience: Africa's policy dilemma	Russo, G.; Bloom, G.; McCoy, D.	commentary	2017	Africa
(140)	Innovative strategies to strengthen health service delivery for universal health coverage in Africa â€“ A scoping literature review	Salawu, M. M.; Onwujekwe, O. E.; Fawole, O. I.	Scoping review	2021	Africa
(141)	Universal Health Coverage and facilitation of equitable access to care in Africa: A systematic review	Yaya, S.; Sanogo, A. N.	systematic review	2019	Africa

(142)	The path towards universal health coverage in the Arab uprising countries Tunisia, Egypt, Libya, and Yemen	Shadi S Saleh, Mohamad S. Alameddine Nabil M. Natafgi Awad Mataria Belgacem Sabri Jamal Nasher Moez Zeiton Shaimaa Ahmad Sameen Siddiqi	Qualitative study	2014	Tunisia, Egypt, Lybia, Yemen
(143)	The role of health service delivery networks in achieving universal health coverage in africa	Simfukwe, K.; Adebisi, Y. A.; Oladunni, A. A.; Mohammed, S. E. E.; Lucero-Prisno, D. E., III	Scoping review	2021	Africa
(144)	Universal health coverage as hegemonic health policy in low- and middle-income countries: A mixed-methods analysis	Smithers, D.; Waitzkin, H.	Mixed methods	2022	LMIC-Africa included
(145)	Editorial: Towards equitable health systems for universal health coverage (UHC) in sub-Saharan Africa	Ataguba, John E.Amporfu, Eugenia Achala, Daniel M. Nabyonga-Orem, Juliet	Editorial	2023	Sub-saharan Africa
(146)	Mandatory health insurance for the informal sector in Tanzania—has it worked anywhere!	Amani Thomas Mori1,2,3	Editorial	2023	Tanzania
(147)	COVID-19 Aftermath: Direction Towards Universal Health Coverage in Low-Income Countries Comment on "Health Coverage and Financial Protection in Uganda: A Political	Tangcharoensathien, V.; Panichkriangkrai, W.; Witthyapipopsakul, W.; Patcharanarumol, W.	case study	2022	Uganda, Ethiopia, Liberia, CAR, Rwanda

	Economy Perspective"				
(148)	Paths towards universal health coverage: Beyond political commitments	Tangcharoensathien, V.; Patcharanarumol, W.; Kulthanmanusorn, A.; Pablos-Mendez, A.	Position paper	2021	LMIC-Africa included
(149)	Analysis of Universal Health Coverage and Equity on Health Care in Kenya	Timothy Chrispinus Okech, Steve Ltumbesi Lelegwe	document analysis	2015	Kenya
(150)	Morocco's policy choices to achieve Universal health coverage	Tinasti, K.	commentary	2015	Morocco
(151)	Identifying the poor for premium exemption: a critical step towards universal health coverage in Sub-Saharan Africa	Umeh, C. A.	Editorial	2017	Sub-saharan Africa
(152)	Challenges toward achieving universal health coverage in Ghana, Kenya, Nigeria, and Tanzania	Umeh, C. A.	Narrative Review	2018	Ghana, Kenya, Nigeria, and Tanzania,
(153)	Strengths and Weaknesses of Strategic Health Purchasing for Universal Health Coverage in Rwanda	Umuhoza, S. M.; Musange, S. F.; Nyandwi, A.; Gatome-Munyua, A.; Mumararungu, A.; Hitimana, R.; Rulisa, A.; Uwaliraye, P.	Mixed methods	2022	Rwanda

(154)	Health care financing in Nigeria: Implications for achieving universal health coverage	Uzochukwu, B. S.; Ughasoro, M. D.; Etiaba, E.; Okwuosa, C.; Envuladu, E.; Onwujekwe, O. E.	Narrative Review	2015	Nigeria
(155)	Structuring Sustainable Universal Health Care in South Africa	Valiani, S.	Narrative Review	2020	South Africa
(156)	Leaving no one behind? Social inclusion of health insurance in low- and middle-income countries: A systematic review	Van Hees, S. G. M.; O'Fallon, T.; Hofker, M.; Dekker, M.; Polack, S.; Banks, L. M.; Spaan, E. J. A. M.	systematic review	2019	LMIC-Africa included
(157)	Community Health Insurance in Low- and Middle-Income Countries	Waelkens, M. P.; Werner, S.; Bart, C.	systematic review	2017	LMIC-Africa included
(158)	The case against labor-tax-financed social health insurance for low- and low-middle-income countries	Yazbeck, A. S.; Savedoff, W. D.; Hsiao, W. C.; Kutzin, J.; Soucat, A.; Tandon, A.; Wagstaff, A.; Yip, W. C. M.	commentary	2020	LMIC-Africa included
(159)	Going universal: how 24 developing countries are implementing universal health coverage from the bottom up	Cotlear, Daniel; Nagpal, Somil; Smith, Owen; Tandon, Ajay; Cortez, Rafael	Report	2015	LMIC-Africa included
(160)	Universal Health Coverage–The Critical Importance of Global Solidarity and Good	Reis, Andreas A	Research article	2016	Global-Africa included

	Governance: Comment on" Ethical Perspective: Five Unacceptable Trade-offs on the Path to Universal Health Coverage				
(161)	Universal health coverage in low-income countries: Tanzaniaâ€™s efforts to overcome barriers to equitable health service access	Wang, Huihui; Rosemberg, Nicolas	Narrative Review	2018	Tanzania
(162)	The Sierra Leone free health care initiative (FHCI): process and effectiveness review	Witter, Sophie; Brikci, Nouria; Harris, Tim; Williams, Richard; Keen, Sarah; Mujica, Ana; Jones, Alex; Murray-Zmijewski, Alex; Bale, Barbara; Leigh, Bailah	Mixed methods	2016	Sierra leon
(163)	Who needs big health sector reforms anyway? Seychellesâ€™ road to UHC provides lessons for Sub-Saharan Africa and Island nations	Workie, N. W.; Shroff, E.; Yazbeck, A. S.; Nguyen, S. N.; Karamagi, H.	Qualitative study	2018	Seychelles
(164)	Realizing Universal Health Coverage in East Africa: The relevance of human rights	Yamin, A. E.; Maleche, A.	Debate	2017	East Africa
(165)	Overcoming distrust to deliver universal health coverage: lessons from Ebola	Woskie, L. R.; Fallah, M. P.	case study	2019	Democratic Republic of Congo

(166)	Tracking universal health coverage: 2021 global monitoring report	World Health Organization	report	2021	Africa included
(167)	Progress on catastrophic health spending in 133 countries: a retrospective observational study	Wagstaff, A.; Flores, G.; Hsu, J.; Smitz, M. F.; Chepynoga, K.; Buisman, L. R.; van Wilgenburg, K.; Eozenou, P.	Quantitative study	2018	LMIC-Africa included

Reference list

1. Kabaniha GA, Afriyie DO, Mandalia ML, Ataguba JE. What do we know about financial protection in health in Africa? A systematic review. 2020.
2. Darrudi A, Khoonsari MHK, Tajvar M. Challenges to achieving universal health coverage throughout the world: a systematic review. Journal of Preventive Medicine and Public Health. 2022;55(2):125.
3. Humphreys G. Gabon gets everyone under one social health insurance roof: Gabon's comprehensive health insurance system is attracting virtually all of its citizens, but to be sustainable it will need to get costs under control. Bulletin of the World Health Organization. 2013;91(5):318-20.
4. Aantjes C, Quinlan T, Bunders J. Towards universal health coverage in Zambia: impediments and opportunities. Development in Practice. 2016;26(3):298-307.
5. Gilbert Abotisem Abiiro GBMMDA. Gaps in universal health coverage in Malawi: A qualitative study in rural communities. BMC Health Services Research. 2014:234.
6. Abraham Assan ATMAAA. Universal health coverage necessitates a system approach: an analysis of Community-based Health Planning and Services (CHPS) initiative in Ghana. Globalization and health. 2018:107.
7. Adewole DA, Osungbade KO. Nigeria National Health Insurance Scheme: a highly subsidized health care program for a privileged few. Int J Trop Dis Health. 2016;19(3):1-11.
8. Admasu K, Balcha T, Ghebreyesus TA. Pro-poor pathway towards universal health coverage: lessons from Ethiopia. J Glob Health. 2016;6(1):010305.
9. Akua Agyepong I, Nana Yaw Abankwah D, Abroso A, ChangBae C, Nii Otoe Dodoo J, Shinye L, et al. The "Universal" in UHC and Ghana's National Health Insurance Scheme: policy and implementation challenges and dilemmas of a lower middle. BMC Health Services Research. 2016;16:1-14.
10. Akhnif EH, Hachri H, Belmadani A, Mataria A, Bigdeli M. Policy dialogue and participation: a new way of crafting a national health financing strategy in Morocco. Health Research Policy and Systems. 2020;18:1-12.
11. Akintoyese Oyekola I, Olusegun Ojediran J, Albert Ajani O, Joseph Oyeyipo E, Rasak B. Advancing alternative health care financing through effective community partnership: A necessity for universal health coverage in Nigeria. Cogent Social Sciences. 2020;6(1):1776946.
12. Alami R. Health financing systems, health equity and universal health coverage in Arab countries. Development and Change. 2017;48(1):146-79.
13. Robert Kaba Alhassan EN-ADKA. A Review of the National Health Insurance Scheme in Ghana: What Are the Sustainability Threats and Prospects? PLoS ONE. 2016:e0165151.

14. Aman A, Gashumba D, Magaziner I, Nordström A. Financing universal health coverage: four steps to go from aspiration to action. The Lancet. 2019;394(10202):902-3.
15. Amani PJ, Hurtig A-K, Frumence G, Kiwara AD, Goicolea I, San SebastiÃ¥n M. Health insurance and health system (un) responsiveness: a qualitative study with elderly in rural Tanzania. BMC Health Services Research. 2021;21(1):1-11.
16. Anib VA, Achiek MM, Ndenzako F, Olu OO. South Sudan's road to universal health coverage: a slow but steady journey. The Pan African medical journal. 2022;42(Suppl 1).
17. Anthony Seddoh FS. Mundane? Demographic characteristics as predictors of enrolment onto the National Health Insurance Scheme in two districts of Ghana. BMC health services research. 2018:330.
18. Appiah B. Universal health coverage still rare in Africa. Can Med Assoc; 2012.
19. Ara I, Zehravi M, Maqbool M, Gani I. A Review of Recent Developments and Future Challenges in the Implementation of Universal Health Coverage Policy Framework in Some Countries. Journal of Pharmaceutical Research & Reports SRC/JPRSR-131 DOI: doi org/1047363/JPRSR/2022 (3). 2022;127.
20. Aregbeshola BS. Enhancing political will for universal health coverage in Nigeria. MEDICC review. 2017;19:42-6.
21. Arie S. Can a low income country move towards universal health coverage? BMJ. 2019;367.
22. Abraham Assan ATMAAA. Challenges to achieving universal health coverage through community-based health planning and services delivery approach: a qualitative study in Ghana. BMJ open. 2019:e024845.
23. Ataguba JE. Assessing equitable health financing for universal health coverage: a case study of South Africa. Applied Economics. 2016;48(35):3293-306.
24. Devi S. Universal health coverage law approved in Egypt. Lancet. 2018;391(10117):194.
25. Naser Derakhshani LDAAAFHS-BVSG. Monitoring Process Barriers and Enablers Towards Universal Health Coverage Within the Sustainable Development Goals: A Systematic Review and Content Analysis. ClinicoEconomics and outcomes research : CEOR. 2020:459-72.
26. Demissie B, Gutema Negeri K. Effect of community-based health insurance on utilization of outpatient health care services in Southern Ethiopia: a comparative cross-sectional study. Risk management and healthcare policy. 2020:141-53.
27. Daff BM, Diouf S, Diop ESM, Mano Y, Nakamura R, Sy MM, et al. Reforms for financial protection schemes towards universal health coverage, Senegal. Bulletin of the World Health Organization. 2020;98(2):100.
28. Chahed MK, Arfa C. Monitoring and evaluating progress towards universal health coverage in Tunisia. PLoS Medicine. 2014;11(9):e1001729.
29. Cashin C, Dossou J-P. Can national health insurance pave the way to universal health coverage in sub-Saharan Africa? Health Systems & Reform. 2021;7(1):e2006122.
30. Namyalo PK, Mutatina B, Byakika S, Walimbwa A, Kato R, Basaza RK. The feasibility analysis of integrating community-based health insurance schemes into the national health insurance scheme in Uganda. Plos one. 2023;18(4):e0284246.
31. Atim C, Bhushan I, Blecher M, Gandham R, Rajan V, Davén J, et al. Health financing reforms for Universal Health Coverage in five emerging economies. Journal of global health. 2021;11.
32. Atinga RA. Healthcare quality under the National Health Insurance Scheme in Ghana: perspectives from premium holders. International Journal of Quality & Reliability Management. 2012.
33. Ayman Fouda FP. Path Dependence and Universal Health Coverage: The Case of Egypt. Frontiers in public health. 2017:325.
34. Barasa E, Rogo K, Mwaura N, Chuma J. Kenya National Hospital Insurance Fund Reforms: implications and lessons for universal health coverage. Health Systems & Reform. 2018;4(4):346-61.

35. Bayarsaikhan D, Tessier L, Ron A. Universal Health Coverage and Social Health Protection: Policy relevance to health system financing reforms. International Social Security Review. 2022;75(2):75-95.
36. Benatar S, Gill S. Universal access to healthcare: the case of South Africa in the comparative global context of the late Anthropocene era. International Journal of Health Policy and Management. 2020;10(2):49.
37. Blecher M, Pillay A, Patcharanarumol W, Panichkriangkrai W, Tangcharoensathien V, Teerawattananon Y, et al. Guest Editorial: Health financing lessons from Thailand for South Africa on the path towards universal health coverage. South African Medical Journal. 2016;106(6):533-4.
38. Borghi J, Maluka S, Kuwawenaruwa A, Makawia S, Tantau J, Mtei G, et al. Promoting universal financial protection: a case study of new management of community health insurance in Tanzania. Health Research Policy and Systems. 2013;11:1-13.
39. Borghi J, Mtei G, Ally M. Modelling the implications of moving towards universal coverage in Tanzania. Health Policy and Planning. 2012;27(suppl_1):i88-i100.
40. Organization WH. Making fair choices on the path to universal health coverage: Final report of the WHO Consultative Group on Equity and Universal Health Coverage. 2014.
41. Makoni M. South Africa passes National Health Insurance Bill. The Lancet. 2023;401(10394):2101.
42. Cerf ME. Health worker resourcing to meet universal health coverage in Africa. International Journal of Healthcare Management. 2021;14(3):789-96.
43. Brikci N. Innovative domestic financing mechanisms for health in Africa: An evidence review. Journal of Health Services Research & Policy. 2024;29(2):132-40.
44. Zaman SB, Hossain N. Universal Health Coverage: A burning need for developing countries. Journal of Medical Research and Innovation. 2017;1(1):18-20.
45. Chemouni B. The political path to universal health coverage: power, ideas and community-based health insurance in Rwanda. World Development. 2018;106:87-98.
46. Chilufya C, Kamanga M. Crunch time: the transformational universal health coverage agenda for Zambia. Health Systems & Reform. 2018;4(4):272-6.
47. Christmals CD, Aidam K. Implementation of the National Health Insurance Scheme (NHIS) in Ghana: lessons for South Africa and low-and middle-income countries. Risk Management and Healthcare Policy. 2020:1879-904.
48. Chuma J, Maina T, Ataguba J. Does the distribution of health care benefits in Kenya meet the principles of universal coverage? BMC public health. 2012;12:1-9.
49. Debie A, Khatri RB, Assefa Y. Contributions and challenges of healthcare financing towards universal health coverage in Ethiopia: a narrative evidence synthesis. BMC health services research. 2022;22(1):866.
50. Monye FN. An appraisal of the national health insurance scheme of Nigeria. Commonwealth Law Bulletin. 2006;32(3):415-27.
51. Dixon J, Tenkorang EY, Luginaah I. Ghana's National Health Insurance Scheme: helping the poor or leaving them behind? Environment and Planning C: Government and policy. 2011;29(6):1102-15.
52. Jane Doherty DKCOJCNEHIKHDM. Does expanding fiscal space lead to improved funding of the health sector in developing countries?: lessons from Kenya, Lagos State (Nigeria) and South Africa. Global health action. 2018:1461338.
53. Domapielle MK. Adopting localised health financing models for universal health coverage in Low and middle-income countries: lessons from the National Health Insurance Scheme in Ghana. Heliyon. 2021;7(6).
54. Dzekem B. Universal Health Coverage in low-and middle-income countries: Lessons learned and way forward.

55. Ekirapa-Kiracho E, Ssennyonjo A, Cashin C, Gatome-Munyua A, Olalere N, Ssempala R, et al. Strategic purchasing arrangements in Uganda and their implications for universal health coverage. Health Systems & Reform. 2022;8(2):2084215.
56. Elhadi YAM, Adebisi YA, Abel UV, Daniel EM, Zaghloul A, Lucero-Prisno D. National health systems strengthening as the primary strategy to achieve Universal Health Coverage in African countries. South Eastern European Journal of Public Health. 2021;2021(2).
57. Eregata GT, Hailu A, Memirie ST, Norheim OF. Measuring progress towards universal health coverage: national and subnational analysis in Ethiopia. BMJ global health. 2019;4(6):e001843.
58. Ezenwaka U, Gatome-Munyua A, Nwankwor C, Olalere N, Orji N, Ewelike U, et al. Strategic health purchasing in Nigeria: investigating governance and institutional capacities within federal tax-funded health schemes and the formal sector social health insurance programme. Health Systems & Reform. 2022;8(2):e2074630.
59. Fenny AP, Yates R, Thompson R. Strategies for financing social health insurance schemes for providing universal health care: a comparative analysis of five countries. Global Health Action. 2021;14(1):1868054.
60. Gajate-Garrido G, Owusua R. The national health insurance scheme in Ghana: Implementation challenges and proposed solutions. 2013.
61. Gonani A, Muula A. Point of View: The importance of Leadership towards universal health coverage in Low Income Countries. Malawi Medical Journal. 2015;27(1):34-7.
62. Grépin KA, Dionne KY. Democratization and Universal Health Coverage: A Case Comparison of Ghana, Kenya, and Senegal. Global Health Governance. 2013;6(2).
63. Hanson K, Barasa E, Honda A, Panichkriangkrai W, Patcharanarumol W. Strategic purchasing: the neglected health financing function for pursuing universal health coverage in low-and middle-income countries: comment on" What's needed to develop strategic purchasing in healthcare? Policy lessons from a realist review". International Journal of Health Policy and Management. 2019;8(8):501.
64. Ipinnimo TM, Durowade KA, Afolayan CA, Ajayi PO, Akande TM. The Nigeria national health insurance authority act and its implications towards achieving universal health coverage. Niger Postgrad Med J. 2022;29(4):281-7.
65. Ismail SA. The rocky road to universal health coverage in Egypt: a political economy of health insurance reform from 2005–15. International Social Security Review. 2018;71(2):79-101.
66. John Koku Awoonor-Williams PTPADHNJA. Does the operations of the National Health Insurance Scheme (NHIS) in Ghana align with the goals of Primary Health Care? Perspectives of key stakeholders in northern Ghana. BMC international health and human rights. 2016:21.
67. Jordi E, Pley C, Jowett M, Abou Jaoude GJ, Haghparast-Bidgoli H. Assessing the efficiency of countries in making progress towards universal health coverage: a data envelopment analysis of 172 countries. BMJ global health. 2020;5(10):e002992.
68. Kelsall T, Hart T, Laws E. Political settlements and pathways to universal health coverage. London: Overseas Development Institute. 2016:1-32.
69. Kenneth Munge SMEWBJC. A Critical Analysis of Purchasing Arrangements in Kenya: The Case of the National Hospital Insurance Fund. International journal of health policy and management. 2017:244-54.
70. Kiendrébéogo JA, De Allegri M, Meessen B. Policy learning and Universal Health Coverage in low-and middle-income countries. Health Research Policy and Systems. 2020;18:1-10.
71. Kiendrébéogo JA, Tapsoba C, Kafando Y, Kaboré I, Sory O, Yaméogo SP. The landscape of strategic health purchasing for universal health coverage in Burkina Faso: insights from five major health financing schemes. Health Systems & Reform. 2022;8(2):2097588.

72. Kiendrébéogo JA, Thoumi A, Mangam K, Touré C, Mbaye S, Odero P, et al. Reinforcing locally led solutions for universal health coverage: a logic model with applications in Benin, Namibia and Uganda. BMJ Global Health. 2021;6(2):e004273.
73. Kigume R, Maluka S. The failure of community-based health insurance schemes in Tanzania: opening the black box of the implementation process. BMC Health Services Research. 2021;21(1):646.
74. Kiri VA, Ojule A. Electronic medical record systems: a pathway to sustainable public health insurance schemes in sub-Saharan Africa. Nigerian Postgraduate Med J 27 (1): 1–7. 2020.
75. Kisangala E, Mbivnjo EL, Okeah BO, Khan MA. Community and informal care providers at the heart of universal health coverage in sub-saharan africa: A position paper. South Eastern European Journal of Public Health. 2023.
76. Koohpayehzadeh J, Azami-Aghdash S, Derakhshani N, Rezapour A, Kalajahi RA, Khasraghi JS, et al. Best practices in achieving universal health coverage: a scoping review. 2020.
77. KUMBURU PN. National health insurance fund (NHIF) in tanzania as a tool for improving universal coverage and accessibility to health care services: Mzumbe University.; 2015.
78. Kutzin J, Yip W, Cashin C. Alternative financing strategies for universal health coverage. World Scientific handbook of global health economics and public policy: volume 1: the economics of health and health systems: World Scientific; 2016. p. 267-309.
79. Mugo MG. The impact of health insurance enrollment on health outcomes in Kenya. Health Economics Review. 2023;13(1):1-19.
80. Laar AS, Asare M, Dalinjong PA. What alternative and innovative domestic methods of healthcare financing can be explored to fix the current claims reimbursement challenges by the National Health Insurance Scheme of Ghana? Perspectives of health managers. Cost Effectiveness and Resource Allocation. 2021;19:1-6.
81. Osetinsky B, Fink G, Kuwawenaruwa A, Tediosi F. Investigating sustainability challenges for the National Health Insurance Fund in Tanzania: a modelling approach. BMJ open. 2023;13(8):e070451.
82. Osei Afriyie D, Kwesiga B, Achungura G, Tediosi F, Fink G. Effects of Health Insurance on Quality of Care in Low-Income Countries: A Systematic Review. Public Health Reviews. 2023;44:1605749.
83. Gina Lagomarsino AGAARMNO. Moving towards universal health coverage: health insurance reforms in nine developing countries in Africa and Asia. Lancet. 2012:933-43.
84. Lavers T. Aiming for universal health coverage through insurance in Ethiopia: state infrastructural power and the challenge of enrolment. Social Science & Medicine. 2021;282:114174.
85. Lay J, Priebe J. “Leave No One Behind” in Middle-Income Countries. A Review of Progress and Policies. NO POVERTY. 2021:129.
86. Letourmy A. [On the development of health insurance in low-income countries: the case of African countries]. Comptes rendus biologies. 2008;331(12):952-63.
87. Lu C, Chin B, Lewandowski JL, Basinga P, Hirschhorn LR, Hill K, et al. Towards universal health coverage: an evaluation of Rwanda Mutuelles in its first eight years. PloS one. 2012;7(6):e39282.
88. Maeda A, Araujo E, Cashin C, Harris J, Ikegami N, Reich MR. Universal health coverage for inclusive and sustainable development: a synthesis of 11 country case studies: World Bank Publications; 2014.
89. Mataria A, El-Saharty S, Hamza MM, Hassan HK. Transforming Health Financing Systems in the Arab World Toward Universal Health Coverage. 2021.
90. Kedar S Mate ALRASGG. Accreditation as a path to achieving universal quality health coverage. Globalization and health. 2014:68.
91. Kedar S Mate ZKSKCFCDCMKTMHSPB. Improving health system quality in low- and middle-income countries that are expanding health coverage: a framework for insurance. International Journal for Quality in Health Care. 2013:497-504.

92. Coveney L, Musoke D, Russo G. Do private health providers help achieve Universal Health Coverage? A scoping review of the evidence from low-income countries. Health Policy and Planning. 2023:czad075.
93. Mathauer I, Musango L, Sibandze S, Mthethwa K, Carrin G. Is universal coverage via social health insurance financially feasible in Swaziland? South African Medical Journal. 2011;101(3):179-83.
94. Mathews C, Goga A, Loveday M, Zembe W, Daviaud E, Siegfried N, et al. Moving towards universal health coverage: Strengthening the evidence ecosystem for the South African health system. South African Medical Journal. 2019;109(11b):8-14.
95. Mbau R, Kabia E, Honda A, Hanson K, Barasa E. Examining purchasing reforms towards universal health coverage by the National Hospital Insurance Fund in Kenya. International journal for equity in health. 2020;19:1-18.
96. Mbindyo R, Kioko J, Siyoi F, Cheruiyot S, Wangai M, Onsongo J, et al. Legal and institutional foundations for universal health coverage, Kenya. Bulletin of the World Health Organization. 2020;98(10):706.
97. McIntyre D. What healthcare financing changes are needed to reach universal coverage in South Africa? SAMJ: South African Medical Journal. 2012;102(6):489-90.
98. McIntyre D, Obse AG, Barasa EW, Ataguba JE. Challenges in financing universal health coverage in sub-Saharan Africa. Oxford research encyclopedia of economics and finance2018.
99. McIntyre D, Van den Heever A. Social or national health insurance: pooling of resources and purchasing of health care. South African health review. 2007;2007(1):71-87.
100. Nabyonga-Orem J, Christmal C, Addai KF, Mwinga K, Aidam K, Nachinab G, et al. The state and significant drivers of health systems efficiency in Africa: A systematic review and meta-analysis. Journal of Global Health. 2023;13.
101. Sen G, Govender V, El-Gamal S. Universal health coverage, gender equality and social protection: A health systems approach. New York (NY) UN Women. 2018.
102. Di McIntyre MKRBKAAH. Promoting universal financial protection: evidence from seven low- and middle-income countries on factors facilitating or hindering progress. Health Research Policy and Systems. 2013:36.
103. Ng'ambi WF, Chigaru F, Mwase T, Banda AJ, Mfutso-Bengo J. Uptake of health insurance in Malawi in 2019-2020: Evidence from the Multiple Indicator Cluster Survey. MedRxiv. 2022:2022.08. 18.22278931.
104. Medcalf AJ, Bhattacharya S, Momen H, Saavedra MA, Jones M. Health for all: The journey to universal health coverage. 2015.
105. Mhazo AT, Maponga CC. The political economy of health financing reforms in Zimbabwe: a scoping review. International journal for equity in health. 2022;21(1):42.
106. Mills A, Ally M, Goudge J, Gyapong J, Mtei G. Progress towards universal coverage: the health systems of Ghana, South Africa and Tanzania. Health policy and planning. 2012;27(suppl_1):i4-i12.
107. Mills A, Ataguba JE, Akazili J, Borghi J, Garshong B, Makawia S, et al. Equity in financing and use of health care in Ghana, South Africa, and Tanzania: implications for paths to universal coverage. The Lancet. 2012;380(9837):126-33.
108. Mitano F, Ventura CAA, Lima MCRAdAd, Balegamire JB, Palha PF. Right to health:(in) congruence between the legal framework and the health system. Revista Latino-Americana de Enfermagem. 2016;24:e2679.
109. Voorhoeve A, Ottersen T, Norheim OF. Making fair choices on the path to universal health coverage: a précis. Health Economics, Policy and Law. 2016;11(1):71-7.
110. Mtei G, Makawia S, Masanja H. Monitoring and evaluating progress towards universal health coverage in Tanzania. PLoS Medicine. 2014;11(9):e1001698.

111. Muinde JVS, Prince RJ. A new universalism? Universal health coverage and debates about rights, solidarity and inequality in Kenya. Soc Sci Med. 2022:115258.
112. Nannini M, Biggeri M, Putoto G. Health coverage and financial protection in Uganda: a political economy perspective. International journal of health policy and management. 2022;11(9):1894.
113. Ndejjo R, Musinguzi G, Musoke D, Wanyenze RK. Unlocking the power of communities to achieve Universal Health Coverage in Africa. South Eastern European Journal of Public Health. 2023.
114. Novignon J, Lanko C, Arthur E. Political economy and the pursuit of universal health coverage in Ghana: a case study of the National Health Insurance Scheme. Health Policy and Planning. 2021;36(Supplement_1):i14-i21.
115. Nshimirimana DA, Kokonya D, Gitaka J, Nzioki JM, Wesonga BO, Mwaura PN, et al. Role of Social Health Insurance towards Universal Health Coverage for East African Community: An Analytical Review.
116. Nyamugira AB, Richter A, Furaha G, Flessa S. Towards the achievement of universal health coverage in the Democratic Republic of Congo: does the Country walk its talk? BMC Health Services Research. 2022;22(1):860.
117. Nyandekwe M, Nzayirambaho M, Kakoma JB. Universal health coverage in Rwanda: dream or reality. The Pan African Medical Journal. 2014;17.
118. Griffith Bell EKMHRJ-HKEOSLLHJKA-WBANAOHLABDS. Assessment of Bypass of the Nearest Primary Health Care Facility Among Women in Ghana. JAMA network open. 2020:e2012552.
119. Obinna Onwujekwe NECMFOHIBUHW. Exploring effectiveness of different health financing mechanisms in Nigeria; what needs to change and how can it happen? BMC health services research. 2019:661.
120. Obi US, Ogbuoji O, Mao W, Shahid M, Onwujekwe O, Yamey G. Progress in the face of cuts: a qualitative Nigerian case study of maintaining progress towards universal health coverage after losing donor assistance. Health Policy and Planning. 2021;36(7):1045-57.
121. Odokonyero T, Mwesigye F, Adong A, Mbowa S. Universal Health Coverage in Uganda: The critical health infrastructure, healthcare coverage and equity. 2017.
122. Okpani AI, Abimbola S. Operationalizing universal health coverage in Nigeria through social health insurance. Nigerian Medical Journal. 2015;56(5):305-10.
123. Onoka CA, Hanson K, Hanefeld J. Towards universal coverage: a policy analysis of the development of the National Health Insurance Scheme in Nigeria. Health policy and planning. 2015;30(9):1105-17.
124. Onoka CA, Onwujekwe OE, Uzochukwu BS, Ezumah NN. Promoting universal financial protection: constraints and enabling factors in scaling-up coverage with social health insurance in Nigeria. Health research policy and systems. 2013;11:1-10.
125. Orem JN, Zikusooka CM. Health financing reform in Uganda: How equitable is the proposed National Health Insurance scheme? International journal for equity in health. 2010;9:1-8.
126. Afriyie DO, Hooley B, Mhalu G, Tediosi F, Mtenga SM. Governance factors that affect the implementation of health financing reforms in Tanzania: an exploratory study of stakeholders' perspectives. BMJ global health. 2021;6(8):e005964.
127. Otieno PO, Asiki G. Making universal health coverage effective in low-and middle-income countries: a blueprint for health sector reforms. Healthcare access-regional overviews. 2020.
128. Owusu-Sekyere E, Bagah DA. Towards a sustainable health care financing in Ghana: Is the National Health Insurance the solution? 2014.
129. Paul E, Deville Cl, Bodson O, SambiÃ©ni NkE, Thiam I, Bourgeois M, et al. How is equity approached in universal health coverage? An analysis of global and country policy documents in Benin and Senegal. International journal for equity in health. 2019;18(1):195.

130. Elisabeth Paul FFRMWvL. Universal Health Coverage in Francophone Sub-Saharan Africa: Assessment of Global Health Experts' Confidence in Policy Options. Global Health: Science and Practice. 2018:260-71.
131. Paul E, Ndiaye Y, Sall FL, Fecher F, Porignon D. An assessment of the core capacities of the Senegalese health system to deliver Universal Health Coverage. Health Policy OPEN. 2020;1:100012.
132. Ranabhat CL, Jakovljevic M, Dhimal M, Kim C-B. Structural factors responsible for universal health coverage in low-and middle-income countries: results from 118 countries. Frontiers in public health. 2020;7:414.
133. Krishna D Rao LPM-GIZCS. Contracting non-state providers for universal health coverage: learnings from Africa, Asia, and Eastern Europe. International journal for equity in health. 2018:127.
134. Rao KD, Petrosyan V, Araujo EC, McIntyre D. Progress towards universal health coverage in BRICS: translating economic growth into better health. Bulletin of the World Health Organization. 2014;92:429-35.
135. Reid M, Gupta R, Roberts G, Goosby E, Wesson P. Achieving Universal Health Coverage (UHC): Dominance analysis across 183 countries highlights importance of strengthening health workforce. PloS one. 2020;15(3):e0229666.
136. Isabelle Agier ALKKSKVrR. Endorsement of universal health coverage financial principles in Burkina Faso. Social Science & Medicine. 2016:157-66.
137. ValÃ©ry Ridde GLHHPJRMDA. Street-level workersÃ¢â‚¬â„¢ inadequate knowledge and application of exemption policies in Burkina Faso jeopardize the achievement of universal health coverage: evidence from a cross-sectional survey. International journal for equity in health. 2018:5.
138. Robert E, Zongo S, Rajan D, Ridde V. Contributing to collaborative health governance in Africa: a realist evaluation of the Universal Health Coverage Partnership. BMC Health Services Research. 2022;22(1):753.
139. Russo G, Bloom G, McCoy D. Universal health coverage, economic slowdown and system resilience: Africa's policy dilemma. BMJ Specialist Journals; 2017. p. e000400.
140. Salawu MM, Onwujekwe OE, Fawole OI. Innovative strategies to strengthen health service delivery for universal health coverage in Africa–a scoping literature review. South Eastern European Journal of Public Health (SEEJPH). 2021.
141. Sanogo NdA, Fantaye AW, Yaya S. Universal health coverage and facilitation of equitable access to care in Africa. Frontiers in public health. 2019;7:102.
142. Shadi S Saleh MSANMNAMBSJNMZSASS. The path towards universal health coverage in the Arab uprising countries Tunisia, Egypt, Libya, and Yemen. Lancet. 2014:368-81.
143. Simfukwe K, Adebisi YA, Oladunni AA, Mohammed SEE, Lucero-Prisno D. The role of health service delivery networks in achieving universal health coverage in Africa. South Eastern European Journal of Public Health. 2021;2021(2).
144. Smithers D, Waitzkin H. Universal health coverage as hegemonic health policy in low-and middle-income countries: A mixed-methods analysis. Social Science & Medicine. 2022;302:114961.
145. Ataguba JE, Amporfu E, Achala DM, Nabyonga-Orem J. Editorial: Towards equitable health systems for universal health coverage (UHC) in sub-Saharan Africa. Frontiers in Health Services. 2023;3 C7 - 1217844.
146. Mori AT. Mandatory health insurance for the informal sector in Tanzania—has it worked anywhere! Frontiers in Health Services. 2023;3.
147. Tangcharoensathien V, Panichkriangkrai W, Witthyapipopsakul W, Patcharanarumol W. COVID-19 Aftermath: Direction Towards Universal Health Coverage in Low-Income Countries Comment on "Health Coverage and Financial Protection in Uganda: A Political Economy Perspective". Int J Health Policy Manag. 2022.

148. Tangcharoensathien V, Patcharanarumol W, Kulthanmanusorn A, Pablos-Mendez A. Paths towards universal health coverage: Beyond political commitments. Journal of Global Health. 2021;11.
149. Timothy Chrispinus Okech SLL. Analysis of Universal Health Coverage and Equity on Health Care in Kenya. Global journal of health science. 2015:218-27.
150. Tinasti K. Morocco's policy choices to achieve Universal health coverage. Pan African Medical Journal. 2015;21(1).
151. Chukwuemeka AU. Identifying the poor for premium exemption: a critical step towards universal health coverage in Sub-Saharan Africa. Global health research and policy. 2017:2.
152. Umeh CA. Challenges toward achieving universal health coverage in Ghana, Kenya, Nigeria, and Tanzania. The International journal of health planning and management. 2018;33(4):794-805.
153. Umuhoza SM, Musange SF, Nyandwi A, Gatome-Munyua A, Mumararungu A, Hitimana R, et al. Strengths and weaknesses of strategic health purchasing for universal health coverage in Rwanda. Health Systems & Reform. 2022;8(2):e2061891.
154. Uzochukwu BS, Ughasoro M, Etiaba Ea, Okwuosa C, Envuladu E, Onwujekwe O. Health care financing in Nigeria: Implications for achieving universal health coverage. Nigerian journal of clinical practice. 2015;18(4):437-44.
155. Valiani S. Structuring sustainable universal health care in South Africa. International Journal of Health Services. 2020;50(2):234-45.
156. Suzanne G M van Hees TOFMHMDSPLMBEJAMS. Leaving no one behind? Social inclusion of health insurance in low- and middle-income countries: a systematic review. International journal for equity in health. 2020:134.
157. Maria-Pia Waelkens and Soors Werner and Criel B. Community Health Insurance in Low- and Middle-Income Countries. In: Stella RQ, editor. International Encyclopedia of Public Health (Second Edition). Second Edition ed. Oxford: Academic Press; 2017. p. 82-92.
158. Yazbeck AS, Savedoff WD, Hsiao WC, Kutzin J, Soucat A, Tandon A, et al. The Case Against Labor-Tax-Financed Social Health Insurance For Low-And Low-Middle-Income Countries: A summary of recent research into labor-tax financing of social health insurance in low-and low-middle-income countries. Health Affairs. 2020;39(5):892-7.
159. Cotlear D, Nagpal S, Smith O, Tandon A, Cortez R. Going universal: how 24 developing countries are implementing universal health coverage from the bottom up: World Bank Publications; 2015.
160. Reis AA. Universal Health Coverage–The Critical Importance of Global Solidarity and Good Governance: Comment on" Ethical Perspective: Five Unacceptable Trade-offs on the Path to Universal Health Coverage". International journal of health policy and management. 2016;5(9):557.
161. Wang H, Rosemberg N. Universal health coverage in low-income countries: Tanzania's efforts to overcome barriers to equitable health service access: World Bank; 2018.
162. Witter S, Brikci N, Harris T, Williams R, Keen S, Mujica A, et al. The Sierra Leone free health care initiative (FHCI): process and effectiveness review. 2016.
163. Workie NW, Shroff E, S. Yazbeck A, Nguyen SN, Karamagi H. Who needs big health sector reforms anyway? Seychelles' road to UHC provides lessons for sub-Saharan Africa and Island nations. Health Systems & Reform. 2018;4(4):362-71.
164. Alicia Ely Yamin AM. Realizing Universal Health Coverage in East Africa: the relevance of human rights. BMC international health and human rights. 2017:21.
165. Woskie LR, Fallah MP. Overcoming distrust to deliver universal health coverage: lessons from Ebola. bmj. 2019;366.
166. Organization WH. Tracking universal health coverage: 2021 global monitoring report: World Health Organization; 2021.

167. Healthcare Access and Quality Index based on mortality from causes amenable to personal health care in 195 countries and territories, 1990-2015: a novel analysis from the Global Burden of Disease Study 2015. Lancet. 2017:231-66.
